# Antidepressant effect of taurine in chronic unpredictable mild stress-induced depressive rats

**DOI:** 10.1038/s41598-017-05051-3

**Published:** 2017-07-10

**Authors:** Gao-Feng Wu, Shuang Ren, Ri-Yi Tang, Chang Xu, Jia-Qi Zhou, Shu-Mei Lin, Ying Feng, Qun-Hui Yang, Jian-Min Hu, Jian-Cheng Yang

**Affiliations:** Liaoning Provincial Key Laboratory of Zoonosis, College of Animal Science & Veterinary Medicine, Shenyang Agricultural University, Shenyang, Liaoning 110866 P.R. China

## Abstract

Depression, a psychiatric and dysthymic disorder, severely affects the learning, work and life quality. The main pathogenesis of depression is associated with central nervous system (CNS) dysfunction. Taurine has been demonstrated to exert protective effects on the brain development and can improve learning ability and memory. Our study investigated the antidepressant-like effects of taurine pre-treatment by examining the changes in depression-like behavior, hormones, neurotransmitters, inflammatory factors and neurotrophic factors in the hippocampus of a chronic unpredictable mild stress (CUMS)-induced depressive rat model. Taurine was found to inhibit the decrease of sucrose consumption and prevent the deficiency of spatial memory and anxiety in rats exposed to CUMS, suggesting a preventive effect of taurine on depression-like behavior. Furthermore, the decreased levels of 5-hydroxytryptamine, dopamine, noradrenaline; the increased levels of glutamate, corticosterone; and the decreased expressions of fibroblast growth factor-2, vascular endothelial growth factor and brain derived neurotrophic factor in depressive rats were hindered by taurine pre-administration. However, tumor necrosis factor-α and interleukin-1β levels were not significantly changed by taurine. The results demonstrated that the anti-depressive effect of taurine may be involved in the regulation of hypothalamic-pituitary-adrenal (HPA) axis and the promotion of neurogenesis, neuronal survival and growth in the hippocampus.

## Introduction

With the rapid development of society and the economy, stress and adversity have become severe and can cause depression, a psychiatric and dysthymic disorder characterized by a wide range of symptoms, such as lasting depression, intellectual ability retardation, cognitive impairment, and somatic symptoms^1^. It has been demonstrated that depression has become a common illness worldwide, affecting approximately 350 million people. According to the WHO’s prediction, the incidence of depression increases 113% per year and is expected to become the world’s second largest disease burden by 2020. Late adolescence or young adulthood is considered a critical period of susceptibility to depression caused by tasks, family problems, transitions and social role changes^2^. Studies have found that exposure to chronic stress during adolescent can lead to changes in endocrine and brain function that may predispose individuals to the development of stress-related psychopathologies, such as depression, and lead to long-term somatic effects in adulthood^3^. What’s more, the pathogenesis of depression is complicated and mainly involves both neuroendocrine and central nervous system (CNS) dysfunction, as well as neurobiological and morphological alterations across several brain regions that are particularly vulnerable to stress, including the hippocampus, which is the main region responsible for learning, memory and emotion^4, 5^. Dysfunction of hippocampus includes neuron reduction, inflammatory stress, hormone release, neurotransmitter system disorder, and the loss of neurotrophic factors^6^. Although many types of anti-depressant drugs such as tricyclic antidepressants (TCAs), monoamine oxidase inhibitors (MAOIs), and selective serotonin reuptake inhibitors (SSRIs) have beneficial effects on depression, most of the currently used anti-depressant drugs have been shown to have different disadvantages, including slow onset, low response rates, toxic effects to organs and drug resistance^7, 8^. Therefore, functional food that could prevent depression and medicine with less or no adverse effects have garnered the attention of researchers.

Taurine, a 2-aminoethylsulfonic acid present in many mammalian tissues, has been reported to participate in various important physiological and pharmaceutical functions, such as tissue structure and function maintenance, membrane stabilization, osmoregulation and neuroprotection^9, 10^. In the brain, taurine presents in the cerebellum, cortex and hippocampus and can be transported into neurons by taurine transporter (TAUT), which is also widely expressed in the CNS, including on the blood–brain barrier (BBB)^11^, indicating that peripheral taurine could be transported into the brain through the BBB and take effect there. Taurine was found to be one of the most important amino acids in the development of immature brains, including maintaining the survival and development of neuronal proliferation, stem cell proliferation and differentiation, and protecting neural cells from excitotoxicity induced by excitatory amino acids^12^. It has also been reported that taurine could increase the emotional learning ability and memory of rats^13^. Furthermore, clinical evidence has demonstrated an increase in the plasma concentration of taurine in depressed patients, while a deficiency in taurine is related to the development of depression, suggesting a close relationship between taurine and depression^14^. Meanwhile, as a semi-essential amino acid of humans, taurine has an observed safe level of supplemental intake in normal healthy adults at up to 3 g/day, and was approved to have no adverse effects for up to 1,000 mg/kg/day by the European Food Safety Authority^15^. Accordingly, taurine may have beneficial effect on depression without adverse effects at a suitable dose, and is a good choice to prevent depression in adolescent or young adulthood.

In the present study, the antidepressant-like effects of taurine given as a pre-treatment were investigated by examining the changes in depression-like behavior, hormones, neurotransmitters, inflammatory factors and neurotrophic factors in the hippocampus of a chronic unpredictable mild stress (CUMS)-induced depressive rat model.

## Results

To investigate the preventive effects of taurine on CUMS-induced depression, late adolescent rats in CUMS stress group (CUMS) were exposed to CUMS every day for 28 days and intraperitoneally injected (i.p.) daily with saline, rats in taurine preventive groups (T1 + CUMS, T2 + CUMS) were exposed to CUMS and i.p. daily with either 200 mg/kg or 500 mg/kg taurine one week prior to the commencement of model establishment, while rats in taurine control (T1, T2) and control groups (Ctr) did not receive any stress, and were i.p. daily with either taurine or the same volume of saline.

### Taurine increased the body weight of CUMS rats

**Figure 1:**
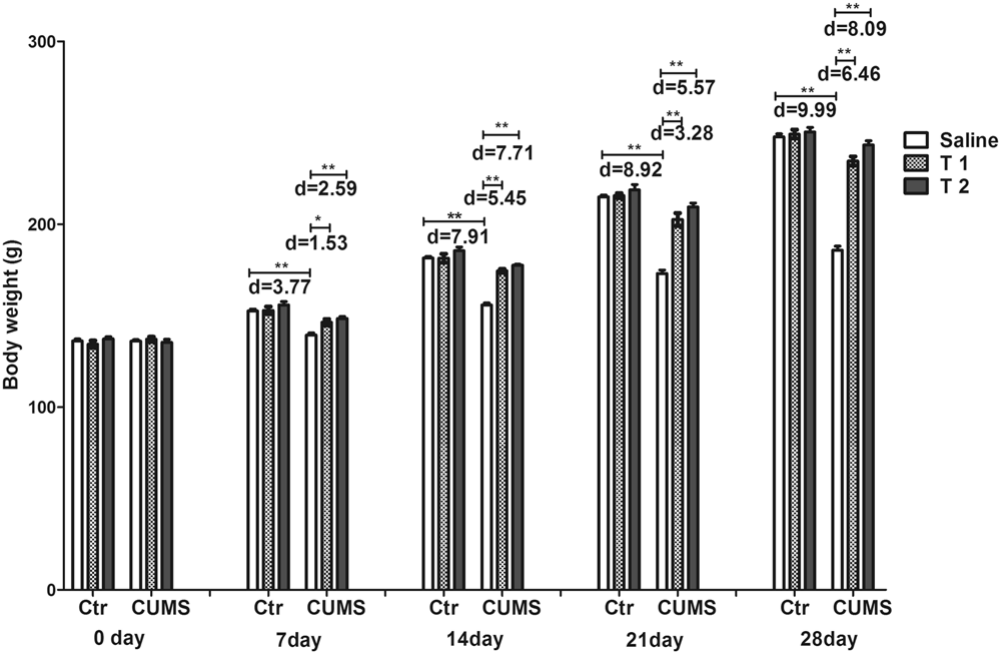
Effects of taurine on the body weight. The body weight was recorded on days 0, 7, 14, 21 and 28 of CUMS were applied. Ctr, control group; CUMS, CUMS group; Saline, i.p. saline; T1, i.p. 200 mg/kg taurine; T2, i.p. 500 mg/kg taurine; All data are expressed as means ± SE and were analyzed by repeated measures ANOVA with group and time as factors followed by Bonferroni’s multiple comparisons post hoc test (n = 10). *P < 0.003 vs. CUMS group; **P < 0.00067 vs. CUMS group. d means Cohen’s d, d = 0.20 was considered as small effect size, d = 0.50 was considered as medium effect size, d = 0.80 was considered as large effect size.

Repeated measures ANOVA revealed a significant interaction between the treatment and week on body weight (F_20, 216_ = 28.68, p < 0.001, f = 1.63). The individual factors week (F_4,216_ = 29430, p < 0.001, f = 7.39) and treatment (F_5,54_ = 190.92, p < 0.001, f = 4.19) also had significant effects. As shown in Fig. 1, no significant differences were observed for the body weight among each group before stress (p > 0.05), but compared with the control, T1 + CUMS and T2 + CUMS groups, the body weight of the CUMS group gained slowly and was remarkably lower on days 7 (p < 0.001, p = 0.002 and p < 0.001 respectively), 14 (p < 0.001), 21 (p < 0.001) and 28 (p < 0.001).

### Taurine reversed the depression-like behavior of CUMS rats

**Figure 2:**
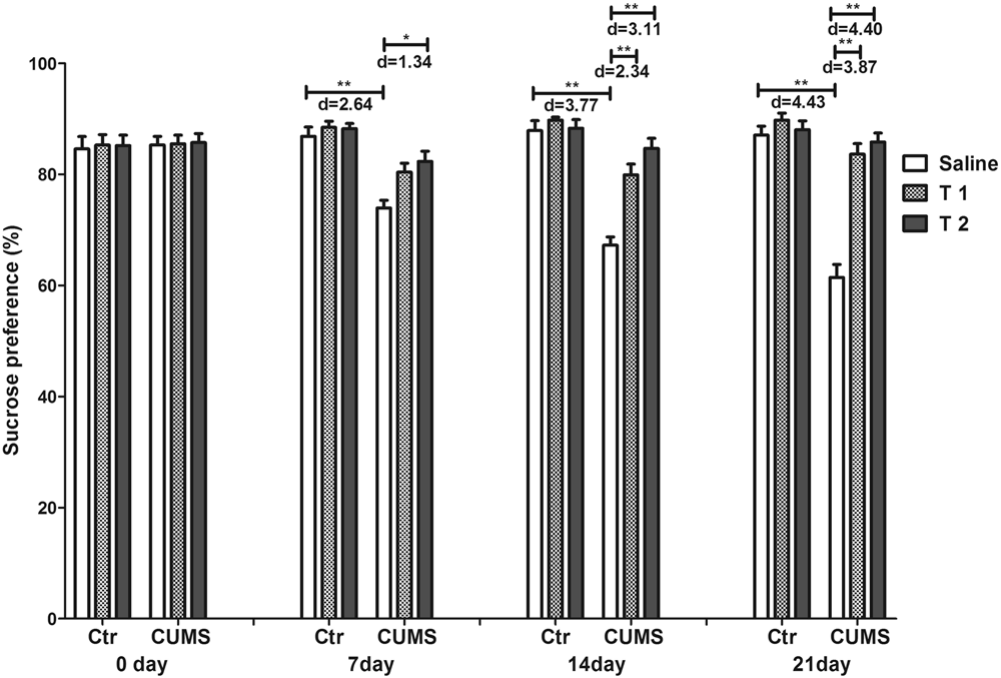
Effects of taurine on sucrose consumption in the SPT. Sucrose intake was recorded on days 0, 7, 14 and 21 of CUMS were applied. Ctr, control group; CUMS, CUMS group; Saline, i.p. saline; T1, i.p. 200 mg/kg taurine; T2, i.p. 500 mg/kg taurine; All data are expressed as means ± SE and were analyzed by repeated measures ANOVA with group and time as factors followed by Bonferroni’s multiple comparisons post hoc test (n = 10). *P < 0.003 vs. CUMS group; **P < 0.00067 vs. CUMS group. d means Cohen’s d, d = 0.20 was considered as small effect size, d = 0.50 was considered as medium effect size, d = 0.80 was considered as large effect size.

Figure 2 showed that the interaction between treatment and week had a significant effect on the sucrose preference test (SPT) (F_15,162_ = 11.68, p < 0.001, f = 1.04). The individual factor treatment (F_5,54_ = 44.16, p < 0.001, f = 2.02) also had significant effects, while the individual factor week did not (F_3,162_ = 2.63, p = 0.052, f = 0.22). All the groups had a similar sucrose consumption baseline on day 0 (p > 0.05), while compared with the control, T1 + CUMS and T2 + CUMS groups, the CUMS rats had lower sucrose preference on days 7 (p < 0.001, p = 0.008 and p = 0.002 respectively), 14 (p < 0.001) and 21 (p < 0.001), indicating that the CUMS group had worsened anhedonia that could be prevented by taurine administration.

**Figure 3:**
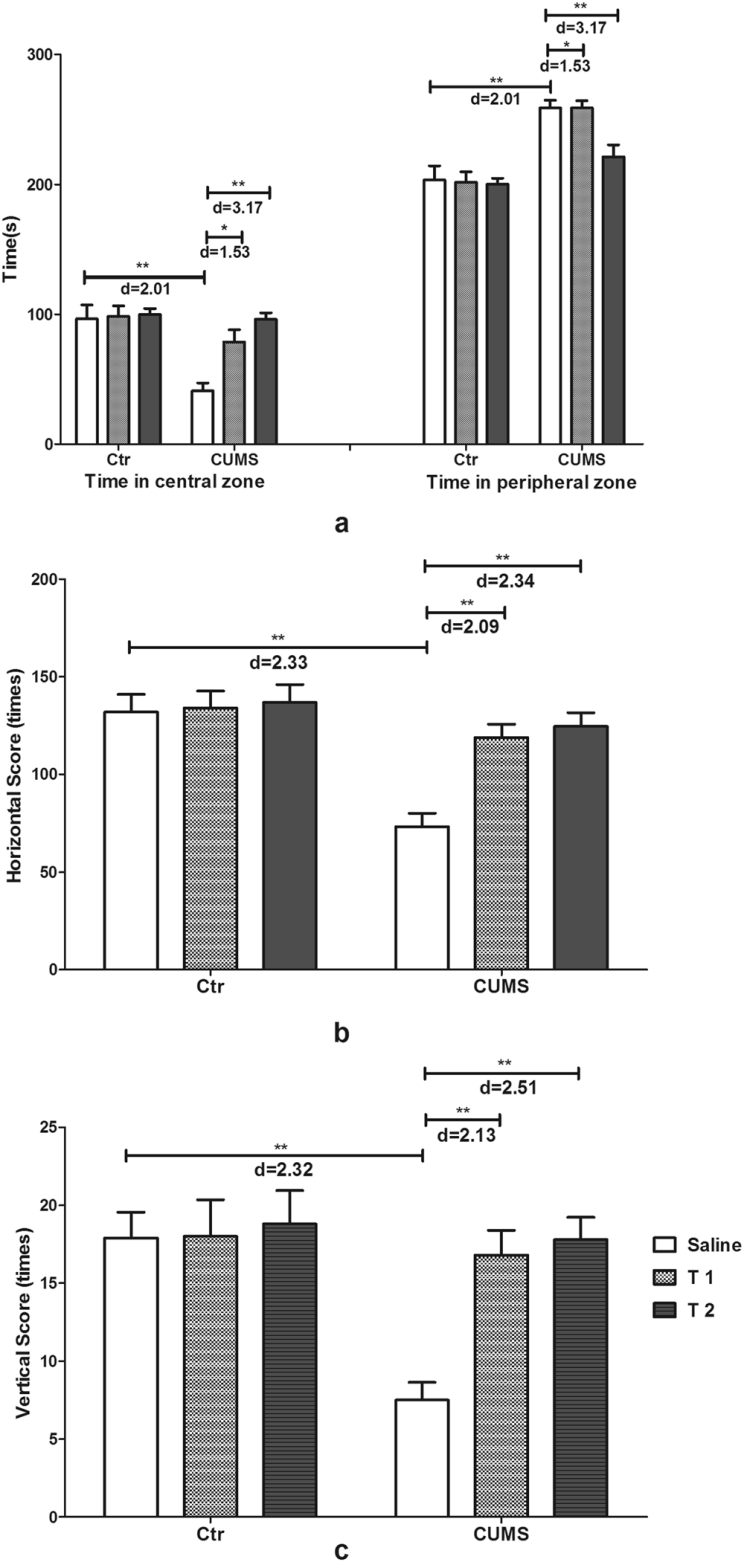
Effects of taurine on the behavior in the OFT. (**a**) Exploration time in the center and margin of rats. (**b**) Horizontal scores of rats in the OFT. (**c**) Vertical scores of rats in the OFT. Ctr, control group; CUMS, CUMS group; Saline, i.p. saline; T1, i.p. 200 mg/kg taurine; T2, i.p. 500 mg/kg taurine; All data are expressed as means ± SE and were analyzed by two-way ANOVA followed by Bonferroni’s multiple comparisons post hoc test (n = 10). *P < 0.003 vs. CUMS group; **P < 0.00067 vs. CUMS group. d means Cohen’s d, d = 0.20 was considered as small effect size, d = 0.50 was considered as medium effect size, d = 0.80 was considered as large effect size.

Figure 3 illustrated the results for the open field test (OFT). Interaction of taurine administration and stress had significant effects on the time in central zone (F_2,54_ = 6.03, p = 0.004, f = 0.47), time in peripheral zone (F_2,54_ = 6.03, p = 0.004, f = 0.48), horizontal score (F_2,54_ = 5.29, p = 0.008, f = 0.44) and vertical score (F_2,54_ = 4.62, p = 0.014, f = 0.41). The main effects of stress significantly affected the time in central zone (F_1,54_ = 17.63, p < 0.001, f = 0.57), time in peripheral zone (F_1,54_ = 17.63, p < 0.001, f = 0.57), horizontal score (F_1,54_ = 19.30, p < 0.001, f = 0.60) and vertical score (F_1,54_ = 8.47, p = 0.005, f = 0.40). The main effects of taurine administration also exerted significant effects on time in central zone (F_2,54_ = 7.67, p = 0.001, f = 0.53), time in peripheral zone (F_2,54_ = 7.67, p = 0.001, f = 0.53), horizontal score (F_2,54_ = 7.13, p = 0.002, f = 0.51) and vertical score (F_2,54_ = 5.79, p = 0.005, f = 0.46). The CUMS group showed a decreased time in the center (time in central zone) with more time spent in the margin (time in peripheral zone) compared with the control, T1 + CUMS and T2 + CUMS groups (p < 0.001, p = 0.001, p < 0.001 respectively). The horizontal and vertical scores of the CUMS group both decreased significantly than the control, T1 + CUMS and T2 + CUMS groups (p < 0.001), indicating that CUMS group had severe anxiety to the new environment, while taurine pre-treated can inhibit the decrease of exploratory behavior and the increase of anxiety of rats exposed to CUMS.

**Figure 4:**
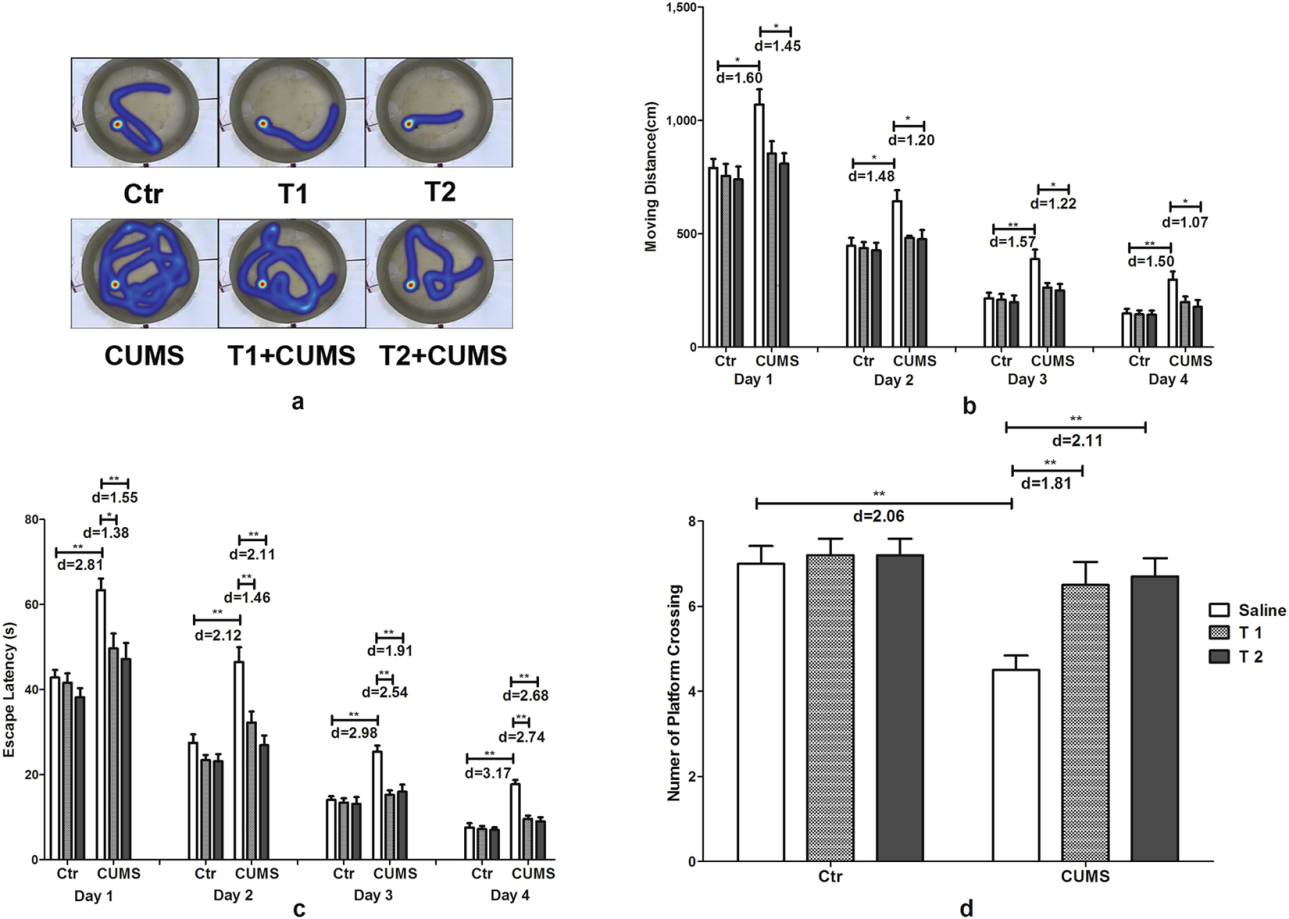
Effects of taurine on behavior in the MWM. (**a**–**c**) Travelled orbit, distances and escape latency of rats in the spatial cognitive ability test. (**d**) Number of platform crosses in the learning and memory test. Ctr, control group; CUMS, CUMS group; Saline, i.p. saline; T1, i.p. 200 mg/kg taurine; T2, i.p. 500 mg/kg taurine; All data are expressed as means ± SE and data in figure b and c were analyzed by repeated measures ANOVA with group and time as factors, data in figure d were analyzed by two-way ANOVA followed by Bonferroni’s multiple comparisons post hoc test (n = 10). *P < 0.003 vs. CUMS group; **P < 0.00067 vs. CUMS group. d means Cohen’s d, d = 0.20 was considered as small effect size, d = 0.50 was considered as medium effect size, d = 0.80 was considered as large effect size.

In the spatial cognitive ability test of the Morris Water Maze (MWM), both day (F_3,162_ = 317.43, p < 0.001, f = 2.43 for moving distance; F_3,162_ = 451.17, p < 0.001, f = 2.89 for escape latency) and treatment (F_5,54_ = 27.76, p < 0.001, f = 1.64 for moving distance; F_5,54_ = 36.29, p < 0.001, f = 1.84 for escape latency) had significant effects, with no significant interaction on moving distance (F_15,162_ = 0.48, p = 0.949, f = 0.21), while had significant interaction on escape latency (F_15, 162_ = 2.16, p = 0.010, f = 0.44). Figure 4 showed that rats in the CUMS group had a longer moving distance than the control and T2 + CUMS groups on day 1 (p = 0.001, p = 0.001 respectively), day 2 (p = 0.001, p = 0.0028 respectively), day 3 (p < 0.001, p = 0.002 respectively) and day 4 (p < 0.001, p = 0.002 respectively), with more escape latency on day 1 than the control, T1 + CUMS and T2 + CUMS groups (p < 0.001, p = 0.001, p < 0.001 respectively), day 2 (p < 0.001), day 3 (p < 0.001) and day 4 (p < 0.001). This finding indicated taurine could prevent the decrease of cognitive ability and memory of non-treated rats exposed to CUMS. Figure 4 also illustrated that the interaction of taurine treatment and stress had a significant effect on the times for platform crossing in the learning memory test of the MWM (F_2,54_ = 4.83, p = 0.012, f = 0.42). The individual factors taurine administration (F_2,54_ = 6.83, p = 0.002, f = 0.50) and stress (F_1,54_ = 11.85, p = 0.001, f = 0.47) both had significant effect. The smallest number of platform crossings was observed in the CUMS group compared with the control, T1 + CUMS and T2 + CUMS groups (p < 0.001), indicating that cognitive ability and memory were impaired by chronic stress, while taurine prevented the decrease of memory in non-treated rats exposed to chronic stress.

### Taurine administration restored the levels of hormones and neurotransmitters in CUMS rats

**Figure 5:**
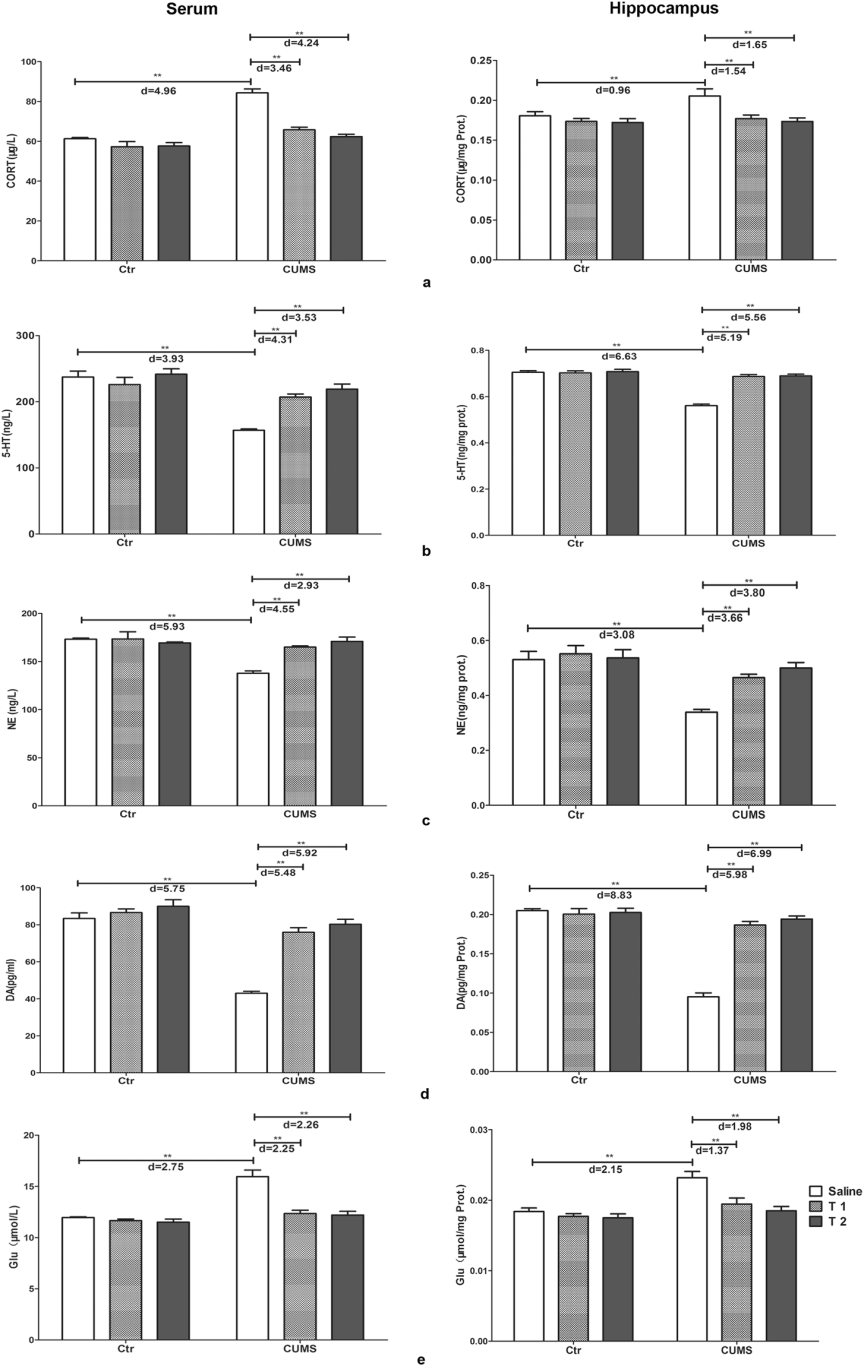
Effects of taurine on the concentrations of CORT and neurotransmitters. (**a**) Concentrations of CORT in the serum and hippocampus. (**b**) Concentrations of 5-HT in the serum and hippocampus. (**c**) Concentrations of DA in the serum and hippocampus. (**d**) Concentrations of NE in the serum and hippocampus. (**e**) Concentrations of Glu in the serum and hippocampus. Ctr, control group; CUMS, CUMS group; Saline, i.p. saline; T1, i.p. 200 mg/kg taurine; T2, i.p. 500 mg/kg taurine; All data are expressed as means ± SE and were analyzed by two-way ANOVA followed by Bonferroni’s multiple comparisons post hoc test (n = 10). *P < 0.003 vs. CUMS group; **P < 0.00067 vs. CUMS group. d means Cohen’s d, d = 0.20 was considered as small effect size, d = 0.50 was considered as medium effect size, d = 0.80 was considered as large effect size.

Serum levels of corticosterone (CORT), neurotransmitters 5-hydroxytryptamine (5-HT), noradrenaline (NE), dopamine (DA) and glutamic acid (Glu) were significantly affected by the interaction of taurine administration and stress (CORT: F_2,54_ = 16.48, p < 0.001, f = 0.781; 5-HT: F_2,54_ = 10.18, p < 0.001, f = 0.61; NE: F_2,54_ = 12.86, p < 0.001, f = 0.69; DA: F_2,54_ = 23.16, p < 0.001, f = 0.93; Glu: F_2,54_ = 13.96, p < 0.001, f = 0.72). The main effects of taurine (CORT: F_2,54_ = 34.68, p < 0.001, f = 1.132; 5-HT: F_2,54_ = 9.52, p < 0.001, f = 0.59; NE: F_2,54_ = 9.45, p < 0.001, f = 0.59; DA: F_2,54_ = 41.53, p < 0.001, f = 1.24; Glu: F_2,54_ = 21.21, p < 0.001, f = 0.89) and stress (CORT: F_1,54_ = 77.57, p < 0.001, f = 1.20; 5-HA: F_1,54_ = 42.19, p < 0.001, f = 0.89; NE: F_1,54_ = 21.10, p < 0.001, f = 0.63; DA: F_1,54_ = 93.42, p < 0.001, f = 1.32; Glu: F_1,54_ = 37.12, p < 0.001, f = 0.83) also had significant effects. Figure 5 illustrated that in the CUMS group serum CORT and Glu were much higher, while serum 5-HT, NE and DA were significantly lower than those in the control group (p < 0.001) and were markedly reversed in the T1 + CUMS and T2 + CUMS groups (p < 0.001).

Hippocampal contents of CORT, 5-HT, NE, DA and Glu were also significantly affected by both taurine (CORT: F_2,54_ = 10.26, p < 0.001, f = 0.62; 5-HT: F_2,54_ = 46.08, p < 0.001, f = 1.31; NE: F_2,54_ = 8.75, p = 0.001, f = 0.57; DA: F_2,54_ = 46.40, p < 0.001, f = 1.31; Glu: F_2,54_ = 10.22, p < 0.001, f = 0.62) and stress (CORT: F_1,54_ = 9.34, p = 0.006, f = 0.42; 5-HT: F_1,54_ = 81.35, p < 0.001, f = 1.23; NE: F_1,54_ = 34.62, p < 0.001, f = 0.80; DA: F_1,54_ = 114.18, p < 0.001, f = 1.45; Glu: F_1,54_ = 22.60, p < 0.001, f = 0.65), with obvious interactions between the two factors (CORT: F_2,54_ = 6.28, p = 0.004, f = 0.48; 5-HT: F_2,54_ = 42.58, p < 0.001, f = 1.26; NE: F_2,54_ = 6.56, p = 0.003, f = 0.49; DA: F_2,54_ = 62.85, p < 0.001, f = 1.53; Glu: F_2,54_ = 4.74, p = 0.013, f = 0.42). Hippocampal CORT and Glu were much higher, 5-HT, NE and DA were much lower in the CUMS group than that of rats in the control group (p < 0.001). In the T1 + CUMS and T2 + CUMS groups, hippocampal CORT and Glu were significantly decreased, while hippocampal 5-HT, NE and DA were obviously increased than those in the CUMS group (p < 0.001). The above results indicated that taurine can obviously inhibited the changes of both serum and hippocampal CORT and neurotransmitters observed in the non-treated rats exposed to CUMS.

### Taurine failed to reverse the levels of inflammatory factors in rats

Significant effects of stress on serum levels of inflammatory cytokines tumor necrosis factor (TNF-α) (F_1,54_ = 17.65, p < 0.001, f = 0.57) and interleukin-1β (IL-1β) (F_1,54_ = 32.02, p < 0.001, f = 0.77) were found. However, no significant effects were observed for taurine administration on serum TNF-α and IL-1β. And the interaction between taurine administration and stress on TNF-α or IL-1β was also not significant. Rats in the CUMS group showed increased serum TNF-α and IL-1β compared with the control group (p < 0.001), with no significant differences compared with rats in either the T1 + CUMS or T2 + CUMS group (P > 0.05).

**Figure 6:**
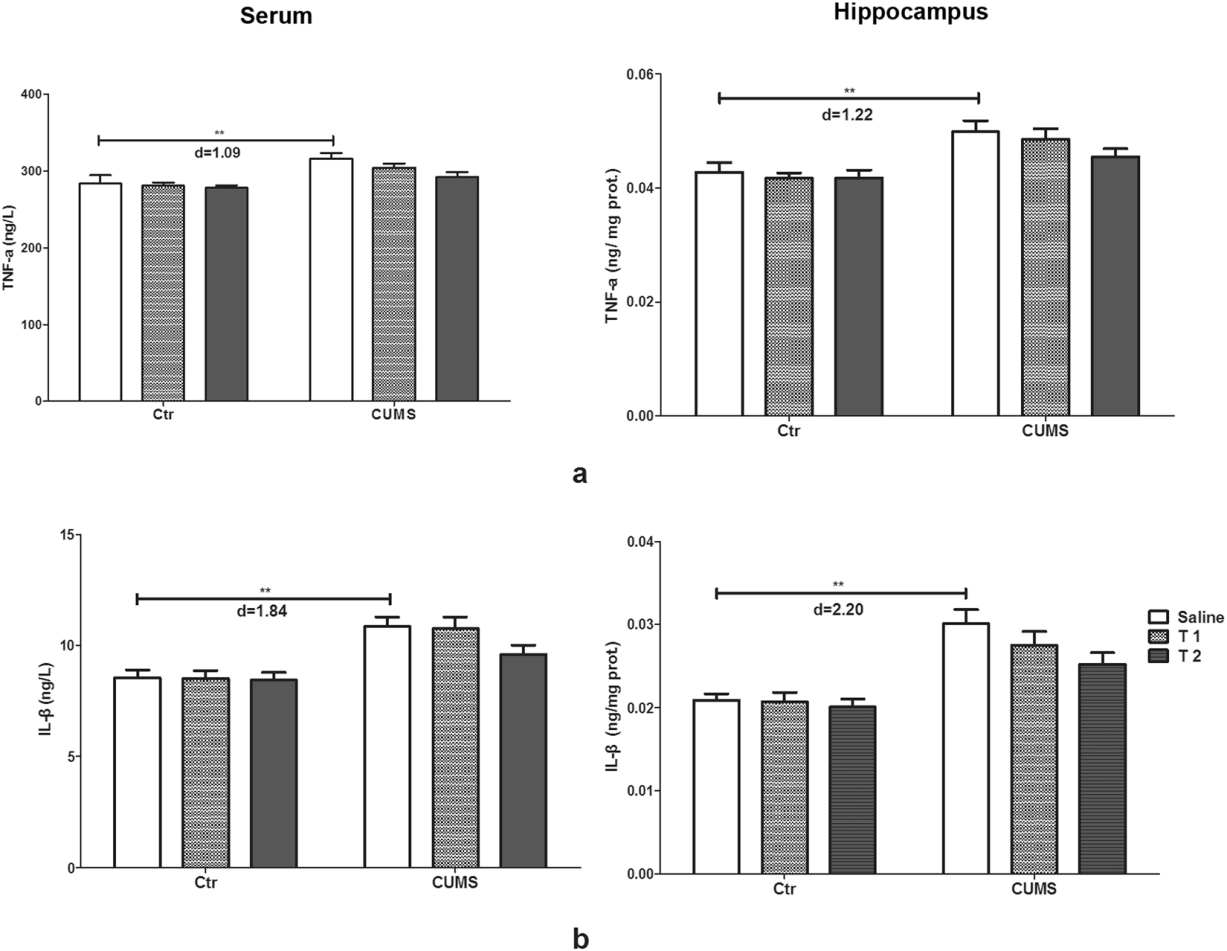
Effects of taurine on the concentrations of cytokines. (**a**) Concentrations of TNF-α in the serum and hippocampus. (**b**) Concentrations of IL-1β in the serum and hippocampus. Ctr, control group; CUMS, CUMS group; Saline, i.p. saline; T1, i.p. 200 mg/kg taurine; T2, i.p. 500 mg/kg taurine; All data are expressed as means ± SE and were analyzed by two-way ANOVA followed by Bonferroni’s multiple comparisons post hoc test (n = 10). * P < 0.003 vs. CUMS group; **P < 0.00067 vs. CUMS group. d means Cohen’s d, d = 0.20 was considered as small effect size, d = 0.50 was considered as medium effect size, d = 0.80 was considered as large effect size.

Significant effects of stress on the hippocampal levels of TNF-α (F_1,54_ = 30.68, p < 0.001, f = 0.75) and IL-1β (F_1,54_ = 43.48, p < 0.001, f = 0.90) were also found. However, no significant effects were observed for taurine administration and its interaction with stress on TNF-α or IL-1β. Both hippocampal TNF-α and IL-1β in the CUMS group were significantly increased compared with the control group (p < 0.001), but had no significant differences compared with rats in either the T1 + CUMS or T2 + CUMS group (P > 0.05), indicating that pre-treatment of taurine at the current doses exerts no significant effects on the inflammation induced by CUMS (Fig. 6).

### Taurine up-regulated the expression of neurotrophic factors in the hippocampus of depressive rats

**Figure 7:**
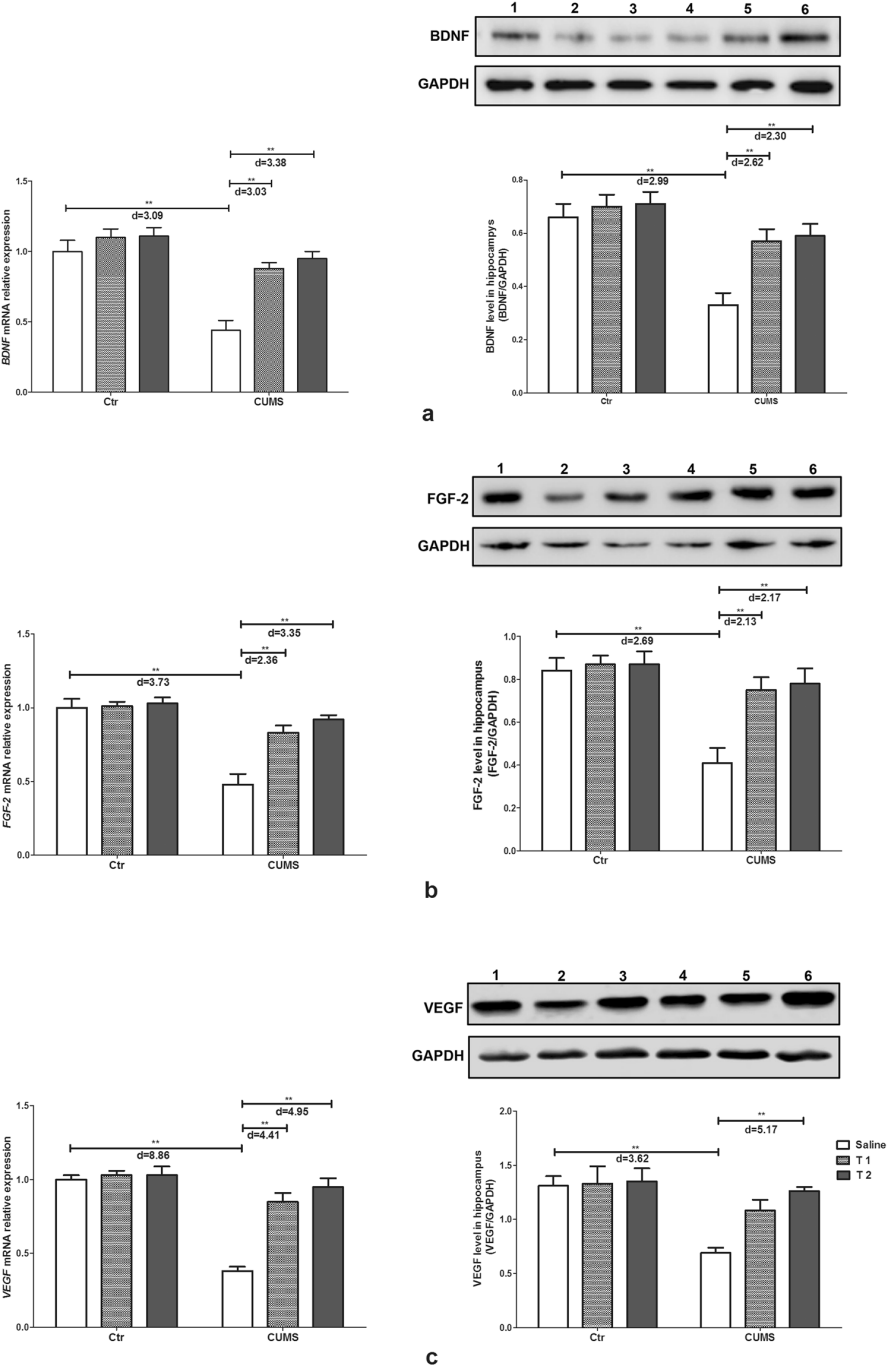
Effects of taurine on the expression of neurotrophic factors in the hippocampus. (**a**) BDNF mRNA and protein expression. (**b**) FGF-2 mRNA and protein expression. (**c**) VEGF mRNA and protein expression. Ctr, control group; CUMS, CUMS group; Saline, i.p. saline; T1, i.p. 200 mg/kg taurine; T2, i.p. 500 mg/kg taurine; In western-blot results, No.1, 2, 3, 4, 5, 6 represent the protein expressions of the control group, CUMS stress group, T1 + CUMS group, T2 + CUMS group, T1 group and T2 group respectively. All data are expressed as means ± SE and were analyzed by two-way ANOVA followed by Bonferroni’s multiple comparisons post hoc test (n = 6). *P < 0.003 vs. CUMS group; **P < 0.00067 vs. CUMS group. d means Cohen’s d, d = 0.20 was considered as small effect size, d = 0.50 was considered as medium effect size, d = 0.80 was considered as large effect size. Full-length blots are presented in Supplementary Figure.

The deficiency of neurotrophic factors is one of the indicative pathogens during depression. In the present study, the interaction between taurine administration and stress exerted significant effects on the gene and protein expression of brain derived neurotrophic factor (BDNF) (F_2,30_ = 6.26, p = 0.005, f = 0.65; F_2, 30_ = 3.83, p = 0.033, f = 0.51), fibroblast growth factor-2 (FGF-2) (F_2,30_ = 10.92, p < 0.001, f = 0.85; F_2,30_ = 5.06, p = 0.013, f = 0.58) and vascular endothelial growth factor (VEGF) (F_2,30_ = 20.08, p < 0.001, f = 1.16; F_2,30_ = 3.75, p = 0.035, f = 0.50). The main effects of taurine and stress also had significant effects on the gene expression of BDNF (F_2,30_ = 15.22, p < 0.001, f = 1.01; F_1,30_ = 39.53, p = 0.002, f = 1.15), FGF-2 (F_2,30_ = 13.24, p < 0.001, f = 0.94; F_1,30_ = 47.73, p < 0.001, f = 1.26), VEGF (F_2,30_ = 24.18, p < 0.001, f = 1.27; F_1,30_ = 62.15, p < 0.001, f = 1.44) and the protein expression of BDNF (F_1,30_ = 27.81, p < 0.001, f = 0.70; F_2,30_ = 7.41, p = 0.002, f = 0.96), FGF-2 (F_1,30_ = 19.72, p < 0.001, f = 0.68; F_2,30_ = 6.85, p = 0.004, f = 0.81) and VEGF (F_1,30_ = 15.55, p < 0.001, f = 0.56; F_2,30_ = 4.76, p = 0.016, f = 0.72). Figure 7 illustrated the decreased mRNA and protein levels of BDNF, FGF-2 and VEGF in the CUMS rats compared with those of rats in the control group (p < 0.001). mRNA expression of these three neurotrophic factors in both the T1 + CUMS and T2 + CUMS groups was up-regulated compared with that in the CUMS group (p < 0.001). Compared with the CUMS group, protein expressions of BDNF, FGF-2 in the T1 + CUMS and T2 + CUMS groups were significantly up-regulated (p < 0.001); protein expression VEGF was only significantly up-regulated in the T2 + CUMS group (p < 0.001). The results indicated that taurine pre-administered could up-regulated expressions of these neurotrophic factors, especially in the T2 + CUMS group.

## Discussion

In the present study, behavioral, biochemical and molecular biological approaches were applied to investigate the preventive effects of taurine on CUMS-induced depression in late adolescent rats. We found that taurine pre-administration produced anti-depressant-like activity and prevented the dysregulation of hormones and neurotransmitters, while inhibited the up-regulation of neurotrophic factors. Depressive rats showed less sucrose intake in the SPT, reduced exploration ability and increased anxiety to the new environment in the OFT, as well as longer escape latency and moving distance in the MWM, which were similar to results from previous studies in both adults and adolescents receiving chronic stress^16, 17^. This finding suggests that a depressive rat model was successfully established in this study. Taurine pre-administered to CUMS rats could significantly inhibit the decrease of sucrose consumption, exploration ability, horizontal score and vertical score in the OFT, and number of platform crossings in the MWM, while hinder the increase of the moving distance and latency in the MWM, indicating an obvious preventive effect of taurine on the depression-like behavior. The results were similar to those from an experiment on young rats, in which taurine exerted important anti-anxiety effects^18^, and also in accordance with the results that taurine pretreatment prevented anesthetic isoflurane-induced cognitive impairment in rats^19^. We also found that taurine supplemented to control rats had no significant effects on behavior, which was in accordance with previous studies that added taurine to normal animals^20^. But Whirley *et al*. found neither antidepressant-like nor anxiolytic-like effects for taurine intraperitoneally injected for only 3 or 8 consecutive days in normal mice^21^, the differences of which may be attributed to two causes. First, the animals did not receive any stress and were all kept under normal conditions. In addition, the duration and dosages of taurine administration, which are essential to revealing the anti-depressant-like effect, were different from those in the present study. Our findings proved an antidepressant-like action of taurine in the classic depressive rat model induced by CUMS.

It has been demonstrated that stress-induced depression is closely associated with disorders of the neuroendocrine system, especially involves in the dysfunction of HPA axis. Clinical and animal experiments proved that chronic stress-induced depression is attributed to the attenuation and disruption of the CORT negative feedback system, which induce the accumulation of CORT in the hippocampus that further damage the structure and function of neurons in the hippocampus by injuring the nutrition unit, lowering the energy metabolism level of neurons, and increasing the vulnerability of neurons to damage, and finally lead to alterations in physiological responsiveness and behavior^22, 23^. During adolescence, which is an important time-point for brain development and active neuroplasticity, important neural pathways involved in stress regulation and HPA axis function are more vulnerable^24^. Studies have indicated an exaggerated and prolonged HPA axis response to stress in adolescents compared to that in adults^25^, and rats exposed to chronic stress during late adolescence are particularly sensitive to somatic and neuroendocrine disorder^26^, indicating that late adolescence may represent a time period of stress hypersensitivity. In the present study, the results that serum and hippocampal CORT in the rats of the CUMS group increased significantly were in accordance with previous studies. While both serum and hippocampal CORT were significantly lower in TI + M and TII + M groups, indicating that taurine pre-administration decreased the over secretion of CORT by regulating HPA under long-term stress, which was similar to the findings of our previous studies on hypertensive rats^27^. The present results on CORT illustrated that taurine could exert its preventive effects on the changes of memory and anxiety behaviors in un-treated rats exposed to CUMS partly through inhibiting neural damage caused by high concentrations of CORT both peripherally and in the hippocampus. However, pre-treatment of taurine at a dosage of 4–7 g/kg/d for only 3 days showed no effects on the blood level of CORT in cold stress rats^28^. The reason may be that in the present study we used different stresses, taurine doses and treatment strategies (i.p. 200 mg and 500 mg/kg/d for 35 days), which could better regulate HPA axis activity.

The “monoaminergic hypothesis” addresses the pathogenesis of depression by stating that depression is probably due to an absolute or relative deficiency in monoamine neurotransmitters including 5-HT, DA and NE^29^. The majority of data support a depletion and dysregultaion of 5-HT, DA and NE in the blood and brain of animals and patients in the back ground of depression. Mutation, reduction and knock out of the receptors of these neurotransmitters in the brain will result in changes in memory, anxiety and reward reaction^30–32^. Moreover, most clinical antidepressants are SSRIs and NE reuptake inhibitors (NRI), which were found to have effects on the treatment of depression in both adults and adolescents^33, 34^. 5-HT, DA and NE could be synthesized both in the periphery and in the brain, among which 5-HT is an intermediate product of tryptophan (Try) metabolism located primarily in peripheral and serotonergic neurons with profuse projections throughout the brain^35^. NE and DA could be synthesized by the adrenal medulla, sympathetic system in the periphery and neurons in the brain, with large numbers of noradrenergic and dopaminergic nerve fibers projecting to the hippocampus to release NE and DA for functions in memory, emotion and reward^36^. Thus, by detecting the levels of 5-HT, DA and NE, one could partly understand the function of the hippocampus. Other neurotransmitters related to depression are amino acid neurotransmitters, especially Glu, an excitatory amino acid. It has been reported that chronic stress and CORT release can increase the accumulation of Glu, which can open the Ca^2+^ channel by binding to N-methyl-D-aspartate receptor (NMDAR) and consequently causes an overload of calcium influx, inducing cytotoxic injury to neurons^37^. Meanwhile, the accumulation of Glu could in turn activate the HPA axis to secrete more GC, which would further strengthen the toxic effects of Glu in the hippocampus^38^. In the present study, neurotransmitters were detected by commercial ELISA kits, although the sensitivities of the kits for Glu, NE, 5-HT, DA were 1 mg/L, 1ng/ml, 1ng/ml and 1 pg/ml respectively, and the intra-assay coefficient of variation were all under 7%, this method was not a exact measurement, as the precursors and the analogues are difficult to be separated from the neurotransmitters, it is usually to get a higher value using ELISA. But because the main purpose of the present study was only to compare the concentrations among different groups, ELISA kits, as a convenient and easy operating method, is a good choice in this study. The results showed that rats in the CUMS group had lower levels of 5-HT, DA, NE, and a higher level of Glu in both the serum and hippocampus compared with rats in the normal control group, the changes of which were in accordance with the previous studies in depressive animal models and patients that decreased circulating or hippocampal levels of 5-HT, NE and DA were detected by ELISA, high-performance liquid chromatography (HPLC) and ultra-high-performance liquid chromatography-tandem mass-spectrometry (HPLC-MS), while Glu level was significantly increased, suggesting that CUMS has a significant effect of monoaminergic and glutamate neurotransmission in brain of rats^39–42^. Other *in vitro* research also found a significant increase in endogenous and K( + )-stimulated serotonin (5-HT) release in the hippocampal slices of depressive rats^43^. The changes in the neurotransmitters of rats exposed to CUMS were found to be significantly hindered by taurine pre-administration in the present study, the effects of which were similar to the clinical antidepressants that enhance the extracelluar 5-HT and the synaptic level of these neurotransmitters in the brain^44^, indicating that taurine may inhibit all the behavior disorders observed in un-treated rats exposed to CUMS by the directly regulation of these neurotransmitters. Further, since HPA hyperfunction could exacerbate the reduction in 5- HT, DA, NE, and the augmentation in Glu^45, 46^, taurine may also indirectly regulate 5- HT, DA, NE and Glu levels through its regulation of HPA activity. Additionally, because 5-HT, DA and NE takes effect in the hippocampus after combined to their receptors, their metabolites and receptors in the hippocampus should be investigated in future studies to illustrate the effects of taurine on the synthesis and secretion of these neurotransmitters.

Considerable evidence has demonstrated that peripheral increase in pro-inflammatory cytokines promoted by chronic stress can cause depression through transmitting immune-mediated signals from the periphery to the CNS^47^. Additionally, in the CNS, cytokines can also be generated by neural cells, microvessel endothelial cells, astrocytes, and microglia in the brain and can induce inflammatory responses affecting neurotransmitter systems to ultimately affect neurocircuits regulating motivation and increased anxiety behaviors relevant to depression^48^. In addition, cytokines can activate the HPA, promote the secretion of CORT and cause damage to neurons in the hippocampus. Our study found an elevation in the concentrations of IL-1β and TNF-α in both the periphery and hippocampus of depressive rats, while taurine pre-administration could slightly lower the levels of cytokines but without significant effects, suggesting that the alleviation effects of taurine on depression-like behavior in the present study were not through its anti-inflammation effects.

Evidence has shown that lacking neurotrophic factors is another pathogenesis of depression. It has been reported that BDNF, FGF-2 and VEGF, which are neurotrophic factor family members that regulate neural proliferation, neurogenesis, apoptosis, expression level of monoamine transmitters, and the function and plasticity of synapses, are closely associated with chronic stress-induced depression both in young adults and adults^49, 50^. It has been reported that under stress and depression conditions, the BDNF, FGF-2 and VEGF levels in the hippocampus decreased^51, 52^, while antidepressant treatment could increase the expression of these neurotrophic factors even to a normal range^53–55^. Meanwhile, depression and anxiety have also been reported to be reversed by BDNF and FGF-2 treatments^56, 57^. The present study observed that taurine pre-treatment significantly inhibited the decrease in the expressions of hippocampal BDNF, FGF-2 and VEGF of rats exposed to CUMS, suggesting that taurine may promote the survival, differentiation, growth and development of neurons in the hippocampus to further protect the neurons from injury and apoptosis through up-regulating BDNF, FGF-2 and VEGF. This result was similar to previous studies that taurine has been found to improve and regulate adult neurogenesis and neurotrophic protein phosphorylation and expression in the hippocampus^58^.

In conclusion, taurine pre-administered to CUMS rats could significantly inhibit the loss of anhedonia, learning ability and memory deficiency, prevent the increase of anxiety to the new environment, hinder the increase of CORT and disorders of neurotransmitters in both serum and hippocampus, prohibit the down-regulation of neurotrophic factors, indicating that taurine has anti-depressant effects and the mechanisms may involved in regulating the HPA function, promoting neurogenesis, cell proliferation, neuronal survival and growth in the hippocampus.

## Methods

### Animals

Seventy two male SPF Wistar rats (120 ± 20 g) obtained from Chang Sheng Biotechnology Co., LTD, Liaoning province, China, were given access to feed and water, and kept at a temperature under 22 ± 2 °C and a 12 hours light and 12 hours dark cycle. Animal handling and experimental procedures followed the Animal Welfare Act and the Guide for the Care and Use of Laboratory Animals and were approved by the Animal Care and Use Committee of Shenyang Agricultural University.

### Experimental procedure

**Figure 8:**
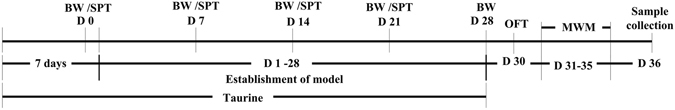
Experimental design. BW: body weight; SPT: sucrose preference test; OFT: open field test; MWM: Morris water maze. Rats were exposed to chronic unpredictable mild stress form day 1 to day 28. Taurine were i.p. daily one week prior to the commencement of model establishment and lasted to day 28. body weight were recorded on days 0, 7, 14, 21 and 28, SPT were carried out on day 0, 7, 14 and 21, OFT was carried out on day 30, MWM was carried out from day 31 to 35, and blood and hippocampus were collected on day 36.

After adaptive feeding, all the rats were divided into 6 groups randomly. Rats in taurine groups (T1, T2, T1 + CUMS, T2 + CUMS) were intraperitoneally injected (i.p.) daily with either 200 mg/kg or 500 mg/kg taurine dissolved in sterile saline, while rats in the control group (Ctr) and CUMS stress group (CUMS) were i.p. the same volume of saline. Taurine was administered to rats one week prior to the commencement of model establishment, which lasted for 35 consecutive days. The experiment was designed as Fig. 8.

### Preparation of CUMS

Rats in the CUMS, T1 + CUMS and T2 + CUMS groups were exposed to CUMS, the procedure of which was designed according to previous studies with some modifications^59^. Rats were kept in a single cage and underwent 28 days of CUMS. The following stressors were used: food deprivation (24 hours), water privation (24 hours), inversion of day/night light cycle (light on in the night and light off in day time), wet bedding (200 ml of water added to 300 g sawdust bedding), clipping the tail with forceps (1 min, the upper 1/3 of the tail), forced swimming (4 °C cold water for 6 min), and electrical stimulation with a voltage of 30–60 V, current of 1 mA and frequency of 2 Hz. The stressors were applied in a completely random order to produce unpredictable mild stress every day.

### Body weight

The body weights of rats were recorded regularly throughout the experiment on days 0, 7, 14, 21 and 28 of CUMS.

### Behavior tests

#### Sucrose preference test (SPT)

SPT was measured one day before and also on the 7^th^, 14^th^ and 21^st^ days of CUMS. There was a 4 days adaption period for rats to habituate to the single cage and consume 1% (w/v) sucrose solution before the test, followed by another 12 hours period of food and water deprivation. Then rats were given 1 h exposure to two identical bottles containing either water or a 1% sucrose solution, which were placed at the same height, randomly on the left or right side of the cages. Both bottles were weighed and recorded before and after the SPT. The SPT value was calculated using the formula: SPT = sucrose intake/ (sucrose intake + water intake) × 100%.

#### Open field test (OFT)

The OFT was performed after CUMS on the day 30. The main apparatus consisted of a square arena (50 × 50 × 70 cm), which was divided into 25 equal squares. In the test, one rat was placed in the center of the arena each time and allowed to explore freely to become acclimated to the environment for 5 minutes. The times that rats spent in the central and peripheral areas were recorded. The number of squares crossed was recorded as the horizontal score, and the number of rearing times was recorded as the vertical score.

#### Morris water maze (MWM)

The MWM was carried out on the 31^st^ day, for which the apparatus and procedures were designed as described by Morris with some modifications^60^. The pool was 150 cm in diameter and 60 cm in height. A round platform was placed in the center of the third quadrant, which was the target quadrant. Warm water (25 ± 1 °C) was added into the pool 2 cm above the platform. In the spatial cognitive ability test, rats were placed into the pool facing the wall, and the time that rats spent finding the platform was recorded as the latency. Rats that failed to find the platform within 120 s were guided and allowed to stay on the platform for 15 s, and the latency was recorded as 120 s. 4 trials were duplicated on each rat per day, over 4 consecutive days. The latency, travel orbit and travel distance were recorded by the EthoVision System 11.0 (Noldus, The Netherlands). The learning and memory test was performed on the 35th day. Each rat was placed in the pool without the platform for 120 s, and the numbers of times that rats crossed the position where the platform was located in the spatial cognitive ability test were recorded by the same Noldus system.

### Sample collection

Blood and hippocampus were collected 24 hours after behavioral tests. Serum was separated after centrifuging at 3000 rpm for 10 minutes at 4 °C, and was kept at −20 °C for further analysis. Hippocampus samples were dissected immediately after the rats were sacrificed, frozen in liquid nitrogen, and then stored under −80 °C.

### Measurement of neurotransmitters, hormones and inflammatory factors

The left hippocampus samples were homogenized using PBS, and the supernatants were collected after centrifuging at 2500 rpm for 10 minutes, at 4 °C. Protein content was determined by BCA protein assay kit (Beyotime, Shanghai, China, P0012S). Serum and hippocampal concentrations of CORT, Glu, NE, 5-HT, DA, IL-1β and TNF-α were analyzed using commercial ELISA kits ((Nuoyuan, Shanghai, China). Serum indexes were detected without any dilution, while hippocampus homogenate were diluted at 1:10 using PBS in duplicate wells. All experimental steps were performed according to the kit specifications. The plates were coated with rat antibodies. The OD values were recorded at 450 nm and the concentrations were calculated with a standard curve. The intra-assay coefficient of variation of CORT, Glu, NE, 5-HT, DA, IL-1β and TNF-α were 5%, 7%, 7%, 5%, 5%, 5% and 5%, respectively. The sensitivities of the assays were 1ng/ml, 1 mg/L, 1ng/ml, 1ng/ml, 1 pg/ml, 1 pg/ml and 1 pg/ml respectively.

### Real-Time PCR analysis

Trizol (Takara, Dalian, China, 9109) was used to extract total RNA from the hippocampus, and reverse transcription was carried out using a commercial kit (Takara, Dalian, China). Real-time PCR carried out at 94°Cfor 5 minutes, and with 32 cycles were run as follows: 94 °C, 30 s; 58.5 °C for *FGF-2*, 63.1 °C for *VEGF*, 60.1 °C for *BDNF*, 30 s; 72 °C, 30 s; and finally terminated at 72 °C, 10 minutes on a Bio-Rad iQTM5 system using SYBR Green PCR Master Mix (ABI). The primers were as follows: *FGF-2* (CCATCAAGGGAGTGTGTGC-forward and GCCCAGTTCGTTTCAGTGC-reverse), *VEGF* (CTGCTGTGGACTTGAGTTGG-forward and CAAACAGACTTCGGCCTCTC-reverse), *BDNF* (CTGCCACTGAAATGCGACT-forward and GCTTCCGAGCCTTCCTTTAG-reverse), *GAPDH* (PRN04, Sangon Biotech, China). Each reaction was repeated three times, and the melting curve was analyzed. The 2^−ΔΔ^Ct method was used to calculate the relative gene expression which was normalized to the expression of the housekeeping gene GAPDH. Data are expressed as the relative fold-change compared with the control group.

### Western Blotting analysis

The proteins of the hippocampus were extracted with a protein extraction kit according to the protocol of the manufacturer (Beyotime, China), and the protein concentration was determined with a BCA protein assay kit (Beyotime, China, P0012S). The proteins were separated with SDS-PAGE electrophoresis and then transferred onto polyvinylidenedifluoride (PVDF) membranes (Bio-Rad, USA). After blocking with 5% (W/V) non-fat milk in Tris buffer solution (TBS) (Applygen, China, B1009) for 2 hours at room temperature, the membranes were first incubated with antibodies against GAPDH (anti mouse GAPDH, Santa Cruze, California, USA, sc-32233, dilution: 1:1000), FGF-2 (anti goat FGF-2, Santa Cruze, California, USA, sc-1390, dilution: 1:1000), BNDF (anti rabbit BDNF, Abcam, Cambridge, UK, ab108319; dilution: 1:1500) and VEGF (anti mouse VEGF, Santa Cruze, California, USA, sc-7269, dilution: 1:500) overnight at 4 °C. After being washed in Tris buffer solution tween (TBST) for 1 hour, the membranes were incubated with appropriate horseradish peroxidase (HRP)-conjugated secondary antibody (1:1000, ZSGB-Bio, China, ZB-2305, ZB-2306, ZB-2301) for 2 hours at room temperature. Signals were visualized by a Super ECL kit (Beyotime, China, P0018). The optical densities of protein bands were recorded by DNR bio-imaging system (Microchemi 4.2, Isreal) and quantified by Image Quant 5.0 software, the relative expressions were normalized to GAPDH.

### Statistical analysis

The data are presented as the mean ± SEM and were analyzed by SPSS 17.0 statistical software (SPSS Inc., Chicago, USA). The distribution of the data was determined by the Komogorov-Smimov test. Between-group comparisons on body weight, SPT and MWM were analyzed by repeated measures ANOVA with group and time as factors followed by Bonferroni’s multiple comparisons post hoc test, which is a safeguard against multiple tests of statistical significance on the same data and alpha was set at 0.05/15 = 0.003 to determine statistical significance. Statistical analyses of the effects of taurine and stress on the OFT, levels of neurotransmitters, hormones, cytokines, mRNA and protein expression of BDNF, FGF-2 and VEGF were carried out using a two- way ANOVA, followed by Bonferroni’s multiple comparisons post hoc test. Effect size of the study was calculated by Cohen’s d and Cohen’s f. Cohen’s d = 0.20 was considered as small effect size, Cohen’s d = 0.50 was considered as medium effect size, Cohen’s d = 0.80 was considered as large effect size; Cohen’s f = 0.10 was considered as small effect size, Cohen’s f = 0.25 was considered as medium effect size, Cohen’s f = 0.40 was considered as large effect size.

## Electronic supplementary material

Figure S1
