# Supplementary material for: Antidepressant effect of taurine in chronic unpredictable mild stress-induced depressive rats
Source: Sci Rep. 2017 Jul 10;7:4989. doi: 10.1038/s41598-017-05051-3 (PMC5504064; doi:10.1038/s41598-017-05051-3)
Supplement: Supplementary file 1 — Figure S1 [file 41598_2017_5051_MOESM1_ESM.pdf]

Supplementary Figure

Antidepressant effect of taurine in chronic unpredictable mild stress-induced depressive rats

Gao-Feng Wu[#], ShuangRen[#], Ri-Yi Tang[#], Chang Xu, Jia-Qi Zhou, Shu-Mei Lin, Ying Feng, Qun-Hui Yang, Jian-Min Hu*, Jian-Cheng Yang*

Liaoning Provincial Key Laboratory of Zoonosis, College of Animal Science & Veterinary Medicine, Shenyang Agricultural University, Shenyang, Liaoning, 110866, P.R. China.

[#] These authors contributed equally to this work

*Corresponding authors: 1. Jian-Min Hu

Fax: 86-88487156

Tel.:86-13066551195

E-mail：hujianmin59@163.com

2. Jian-Cheng Yang

Fax: 86-88487156

Tel.:86-15040040167

E-mail：syauyjc@126.com

Liaoning Provincial Key Laboratory of Zoonosis

College of Animal Science & Veterinary Medicine

Shenyang Agricultural University

Shenyang, Liaoning, 110866, P.R. China

BDNF (15 KDa)

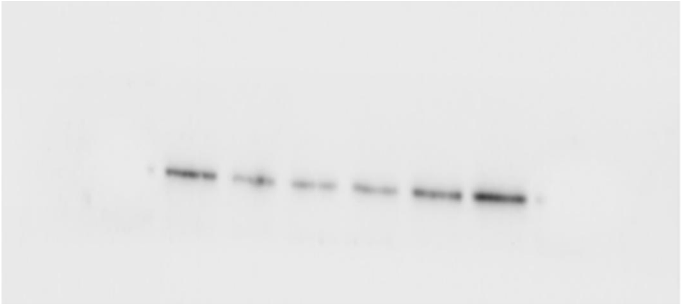

a

GAPDH (37 KDa)

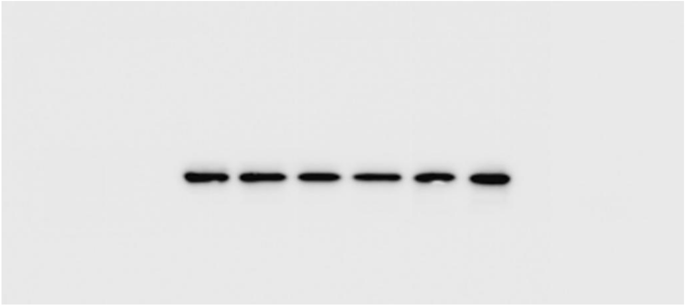

b

FGF-2 (24 KDa)

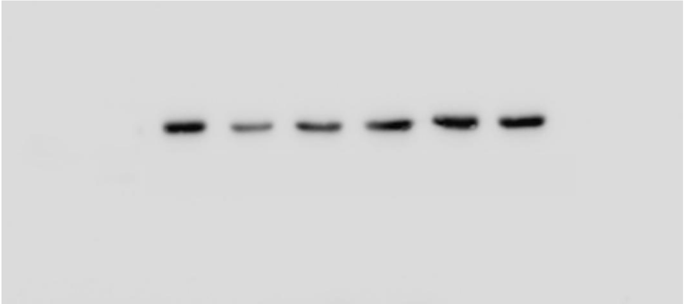

C

GAPDH (37 KDa)

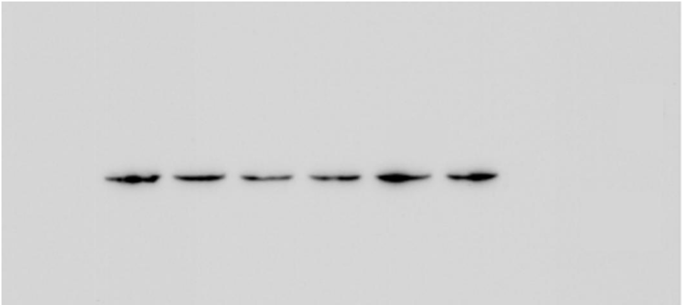

d

VEGF (21 KDa)

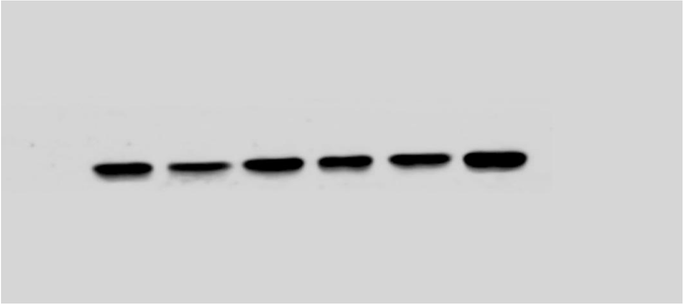

e

GAPDH (37 KDa)

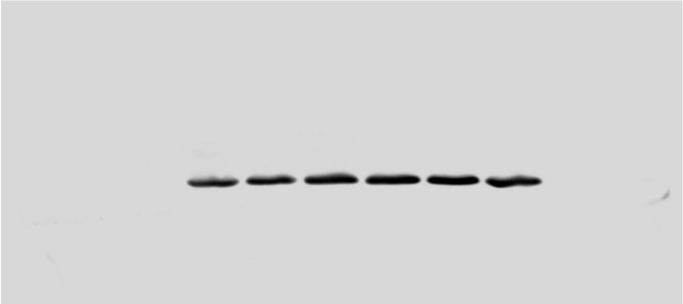

f

Figure S1. Original blots of BDNF, FGF-2, VEGF and GAPDH. After the successful transfer of protein on PVDF membranes, membranes were blocked and eventually cropped horizontally according to the desired protein of interest. Thereafter each membrane was processed for respective antibody incubation and detection as described in method section of main text.
